# Prophylactically injection of Nicorandil to reduce no-reflow phenomenon during PCI in acute STEMI patients: Protocol of a double-blinded, randomized, placebo-controlled trial

**DOI:** 10.1097/MD.0000000000025500

**Published:** 2021-04-16

**Authors:** Su An, Huopeng Huang, Huaying Wang, Yunlu Jiang

**Affiliations:** Chongqing Dazu District People's Hospital, Chongqing, China.

**Keywords:** Nicorandil, no-reflow phenomenon, percutaneous coronary intervention, ST-elevation myocardial infarction

## Abstract

**Introduction:**

An acute ST-elevation myocardial infarction (STEMI) is a very serious type of heart attack and a profoundly life-threatening medical emergency, and percutaneous coronary intervention (PCI) is the preferred strategy. However, in patients undergoing primary PCI, 30% to 40% may suffer the no-reflow phenomenon (NRP), and it could expand the myocardial infarction area and accompanied with high rehospitalization rate and fatality rate. In this study, we try to conduct a double blinded, randomized, placebo-controlled trial to observe whether the prophylactically intracoronary administration of Nicorandil could reduce the occurrence of NRP in STEMI patients undergoing PCI.

**Methods:**

Simple randomization in a 1:1 ratio will be made in blocks of variable size according to a random numbers generated by Excel 2010 to divide the patients to treatment group (Nicorandil) and control group (Saline). The outcomes are the occurrence of NRP, levels of interleukin-6 and HS-CRP, cTnT, and CK-MB before, and every 4 hours following PCI, and major adverse cardiovascular events at day 30. SPSS 23.0 (IBM, Chicago, IL) will be used, and *P*-value < .05 will be considered statistically significant.

**Conclusions:**

The findings will determine the efficacy of prophylactically intracoronary administration of Nicorandil to reduce the occurrence of NRP during PCI in acute STEMI patients.

**Trial registration:**

OSF Registration number: DOI 10.17605/OSF.IO/QPF3V

## 1 Introduction

An acute ST-elevation myocardial infarction (STEMI) is a very serious type of heart attack and a profoundly life-threatening medical emergency, cause by the occlusion of 1 or more coronary arteries to result in myocardial injury or necrosis. Approximately 500,000 patients suffer STEMI annually,^[1]^ and it is estimated that STEMI comprises 25% to 40% of myocardial infarction presentation.^[2]^ Reperfusion therapy is reasonable for STEMI 12 to 24 hours within the onset. Percutaneous coronary intervention (PCI) is the preferred strategy, and it could rapidly re-canalize the infarct arteries, significantly reduce infarct area, and improve clinical outcome and long-term prognosis.^[3,4]^

However, in patients undergoing primary PCI, although without obvious dissection, thrombosis, spasm, or severe residual stenosis, 30% to 40% of patients may suffer the no-reflow phenomenon (NRP)^[5,6]^: after the recanalization of infarcted arteries, coronary blood flow to the ischemic tissue may still be impeded and the myocardial cell perfusion cannot be maintained. NRP is one of the common serious complications after PCI in STEMI patients, and it could expand the myocardial infarction area and accompanied with high rehospitalization rate and fatality rate.^[7–9]^

Many efforts have been made to prevent or reduce the occurrence of NRP, such as the administration of statins and Nicorandil.^[10–12]^ Nicorandil is an ATP-sensitive K^+^ opener, and it could dilate coronary artery to increase blood flow. Nicorandil has been administered in treating NRP in patients after PCI in some studies, and it may improve the symptoms of no-reflow in patient.^[13]^ However, there is still a lack of high quality randomized controlled trial to show the efficacy and safety of Nicorandil in preventing NRP during PCI in acute STEMI patients. In this study, we try to conduct a double blinded, randomized, placebo-controlled trial to observe whether the prophylactically intracoronary administration of Nicorandil could reduce the occurrence of NRP in STEMI patients undergoing PCI.

## 2 Methods

### 2.1 Study design

**Figure 1:**
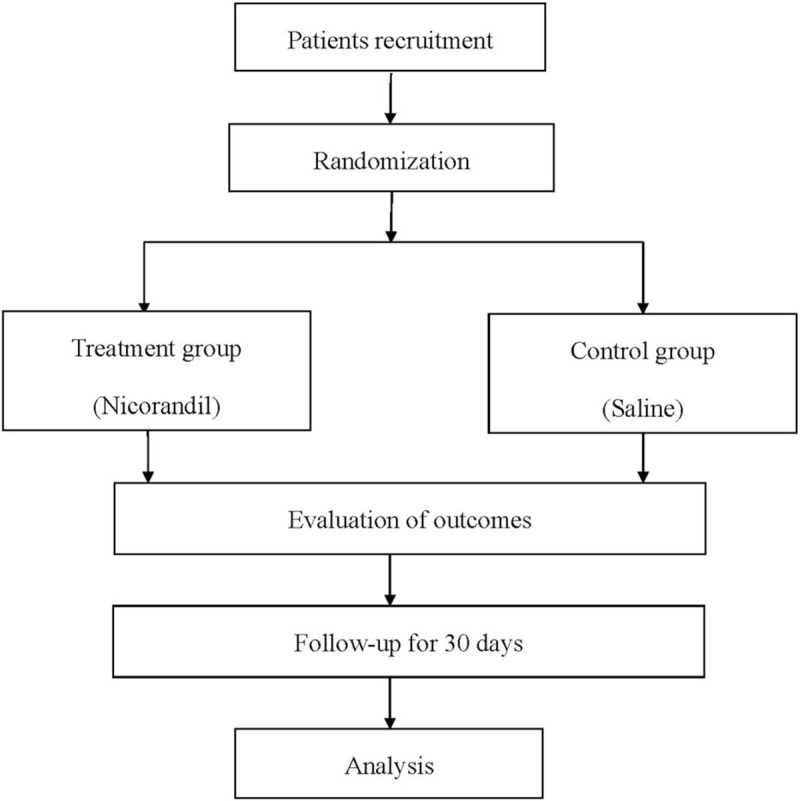
Flow diagram of the study.

We will perform the double blinded, randomized, placebo-controlled trial (registration number: DOI 10.17605/OSF.IO/QPF3V) in Chongqing Dazu District People's Hospital from May April 1st, 2021 to April 31st, 2022. The protocol has been approved by the Health Research Ethics Board in the hospital. We will carry out the procedure in accordance with the Declaration of Helsinki. The flow of patients screening, randomization, and follow-up are presented in Figure 1.

### 2.2 Participants studied

Patients with sustained acute STEMI and suitable for PCI in hospital will be screened. Patients meeting the following criteria will be included:

(1) aging from 18 to 85 years old;
(2) diagnosis of acute STEMI within 12 hours;
(3) total occlusion of the infarcted artery;
(4) blood pressure less than 90/60 mm Hg.

Patients with left bundle-branch block, kidney dysfunction, and histories of myocardial infarction, PCI, coronary artery bypass grafting will be excluded. Before randomization, all patients or authorized persons will sign a written informed consent, and they can freely choose whether to continue the trial at any time.

### 2.3 Randomization and blinding

Simple randomization in a 1:1 ratio will be made in blocks of variable size according to a random numbers generated by Excel 2010 to divided the patients to treatment group (intracoronary injection of 2 mg Nicorandil) and control group (intracoronary injection of 2 mL Saline). The attending physician, patients, outcome evaluators, and statistical analysts will be blinded to the allocation. To maintain blinding, Nicorandil and Saline are in the same package and appearance.

### 2.4 Interventions

All patients in both groups will take antiplatelet drugs including 300 mg Aspirin (Bayer, Germany) and 600 mg Clopidogrel (Sanofi [Hangzhou] Pharmaceutical Co., Ltd, Hangzhou, China), and Heparin (70 U/kg) will be injected through the artery sheath tube before PCI.

In the treatment group, 2 mg Nicorandil (Beijing Sihuan Kebao Pharmaceutical Co. Ltd, Beijing, China) will be injected 2 mm distal to the site of the occlusion in the coronary artery in the thrombus-aspiration catheter. Angiography will be repeated 5 minutes later to determine whether to repeatedly administer 2 mg Nicorandil. The total dose of Nicorandil should not exceed 6 mg in any patients.

In the control group, 2 mL Saline (Tianjin Barence Biotechnology Co., Ltd, Tianjin, China) will be injected 2 mm distal to the site of the occlusion in the coronary artery in the thrombus-aspiration catheter. Angiography will be repeated 5 minutes later to determine whether to repeatedly administer 2 mL Saline. The total dose of Nicorandil should not exceed 6 mL in any patient.

### 2.5 Outcome measures

The primary outcome is the occurrence of NRP defined as follows: thrombolysis in myocardial infarction flow grade less than 2 without dissection, spasm, or significant residual stenosis, at the end of interventional procedure, and a decrease of less than 50% of the basal elevation of ST segment at 90 minutes after reperfusion with PCI.

The secondary outcomes are levels of interleukin-6 and HS-CRP, cTnT, and CK-MB before, and every 4 hours following PCI, and major adverse cardiovascular events (postinfarction angina, reinfarction, heart failure, arrythmias, cardiogenic shock, and cardiovascular death) at day 30.

### 2.6 Sample size

As previous literature reports, the occurrence of NRP was over 30%.^[5,6]^ We preliminarily found that Nicorandil could reduce the occurrence to 15%, and taking α = 5%, 1-β = 80% with a 2-sided test, 118 cases will be calculated in each group. Considering a potential dropout of 20% in the 30-day-follow-up, 280 cases will be needed in total.

### 2.7 Statistical methods

Data with a normal distribution will be expressed as mean ± standard deviation, and categorical data as percentages. Student *t* test, Wilcoxon rank-sum test, Mann–Whitney *U* test, *χ*^2^ test, or Fisher exact test will be applied accordingly. SPSS 23.0 (IBM, Chicago, IL) will be used, and *P* < .05 will be considered statistically significant.

## 3 Discussion

Currently, PCI is considered as one of the most effective measures for the treatment of acute STEMI.^[4,14,15]^ Within 12 hours of the onset of STEMI, PCI could rapidly re-canalize the infarcted related arteries, restore myocardial perfusion, and significantly reduce infarct size and mortality in acute STEMI patients. However, coronary angiography studies showed that a large number of patients suffer from NRP, the inadequate reperfusion of the myocardium without evidence of coronary artery obstruction after PCI.^[5,6]^ The phenomenon was thought to be associated with endothelial damage, inflammation, vascular spasm, platelet activation and aggregation, and thromboembolism.^[16,17]^ The NRP can be a marker of myocardial injury, ischemia, infarction, and a predictor of higher incidence of major adverse cardiovascular events. It could seriously affect the prognosis of STEMI patients and increase mortality, which makes it in great significance to prevent and reduce the occurrence of NRP in STEMI patients during PCI.

It has been reported that Nicorandil could improve endothelia, inflammation, fibrinolytic ability, and stabilize plaques,^[18–20]^ and the prophylactic application of Nicorandil before PCI may have the potential to reduce the occurrence of NRP to improve prognosis for acute STEMI. Therefore, to provide higher clinical evidence, we try to conduct a double blinded, randomized, placebo-controlled trial to determine the efficacy of prophylactically intracoronary administration of Nicorandil to reduce the occurrence of NRP during PCI in acute STEMI patients.

Indeed, several limitations need to be addressed. First, there might be bias due to the single-centered study. Second, quantitative analyses for accurately evaluating cardiac function are also needed, such as SPECT and cardiac magnetic resonance.

## Author contributions

**Data curation:** Su An, Huopeng Huang.

**Funding acquisition:** Yunlu Jiang.

**Investigation:** Yunlu Jiang.

**Resources:** Su An, Huopeng Huang.

**Software:** Huopeng Huang, Huaying Wang.

**Supervision:** Huaying Wang.

**Writing – original draft:** Su An, Huopeng Huang.

**Writing – review & editing:** Su An, Yunlu Jiang.
